# SARS-CoV-2 Spike Protein Stabilized in the Closed State Induces Potent Neutralizing Responses

**DOI:** 10.1128/JVI.00203-21

**Published:** 2021-07-12

**Authors:** George W. Carnell, Katarzyna A. Ciazynska, David A. Wells, Xiaoli Xiong, Ernest T. Aguinam, Stephen H. McLaughlin, Donna Mallery, Soraya Ebrahimi, Lourdes Ceron-Gutierrez, Benedikt Asbach, Sebastian Einhauser, Ralf Wagner, Leo C. James, Rainer Doffinger, Jonathan L. Heeney, John A. G. Briggs

**Affiliations:** a Laboratory of Viral Zoonotics, Department of Veterinary Medicine, University of Cambridge, Cambridge, United Kingdom; b Medical Research Council Laboratory of Molecular Biology, Cambridge, United Kingdom; c DIOSynVax, University of Cambridge, Cambridge, United Kingdom; d Department of Clinical Biochemistry and Immunology, Addenbrooke’s Hospital, Cambridge, United Kingdom; e Institute of Medical Microbiology and Hygiene, University of Regensburg, Regensburg, Germany; f Institute of Clinical Microbiology and Hygiene, University Hospital Regensburg, Regensburg, Germany; Cornell University

**Keywords:** SARS-CoV-2, glycoproteins, immunization, neutralizing antibodies

## Abstract

The majority of SARS-CoV-2 vaccines in use or advanced development are based on the viral spike protein (S) as their immunogen. S is present on virions as prefusion trimers in which the receptor binding domain (RBD) is stochastically open or closed. Neutralizing antibodies have been described against both open and closed conformations. The long-term success of vaccination strategies depends upon inducing antibodies that provide long-lasting broad immunity against evolving SARS-CoV-2 strains. Here, we have assessed the results of immunization in a mouse model using an S protein trimer stabilized in the closed state to prevent full exposure of the receptor binding site and therefore interaction with the receptor. We compared this with other modified S protein constructs, including representatives used in current vaccines. We found that all trimeric S proteins induced a T cell response and long-lived, strongly neutralizing antibody responses against 2019 SARS-CoV-2 and variants of concern P.1 and B.1.351. Notably, the protein binding properties of sera induced by the closed spike differed from those induced by standard S protein constructs. Closed S proteins induced more potent neutralizing responses than expected based on the degree to which they inhibit interactions between the RBD and ACE2. These observations suggest that closed spikes recruit different, but equally potent, immune responses than open spikes and that this is likely to include neutralizing antibodies against conformational epitopes present in the closed conformation. We suggest that closed spikes, together with their improved stability and storage properties, may be a valuable component of refined, next-generation vaccines.

**IMPORTANCE** Vaccines in use against SARS-CoV-2 induce immune responses against the spike protein. There is intense interest in whether the antibody response induced by vaccines will be robust against new variants, as well as in next-generation vaccines for use in previously infected or immunized individuals. We assessed the use as an immunogen of a spike protein engineered to be conformationally stabilized in the closed state where the receptor binding site is occluded. Despite occlusion of the receptor binding site, the spike induces potently neutralizing sera against multiple SARS-CoV-2 variants. Antibodies are raised against a different pattern of epitopes to those induced by other spike constructs, preferring conformational epitopes present in the closed conformation. Closed spikes, or mRNA vaccines based on their sequence, can be a valuable component of next-generation vaccines.

## INTRODUCTION

The surface of the SARS-CoV-2 virion is studded with spike (S) protein trimers. They are predominantly in a prefusion form (1, 2) in which the three copies of the receptor binding domain (RBD) are located at the top of the spike, surrounded by the N-terminal domains (NTDs) (3, 4). Prefusion S trimers are in a stochastic mixture of conformations (1), either closed, in which all three RBDs lie down at the top of the spike, or open, in which one or more of the RBDs protrude from the top of the spike. The receptor binding site (RBS) on the RBD which is responsible for interaction with the receptor ACE2 is largely occluded when the RBD is in the down position (3–6). S contains a furin cleavage site, at which it can be separated into S1 and S2 subunits. Cleavage modulates infectivity in a cell type-dependent manner (7).

After interaction with the receptor, S undergoes a conformational rearrangement leading to exposure of S2, insertion of the fusion peptide (FP) into the membrane of the target cell, and refolding of S2 into the elongated postfusion form (8). This refolding pulls the fusion peptide and transmembrane domain of S together, drawing the target cell and viral membranes together and causing their fusion.

The currently licensed SARS-CoV-2 vaccines in clinical use are designed to deliver the first-sequenced 2019 B lineage S protein of SARS-CoV-2 as the immunogen (9). The first three licensed vaccines require delivery of either mRNA (Pfizer/BioNTech [10] and Moderna [11]) or attenuated chimpanzee adenovirus (AstraZeneca/Oxford [12]) for the expression of S protein by target cells *in vivo*. Vaccine candidates based on inactivated virus, VLP, or recombinant protein present the S antigen directly. Since the goal is to generate neutralizing antibodies against the virus, a number of the vaccines rely on mutations in S to stabilize the prefusion state to reduce stochastic transition into the postfusion form. Mutations incorporated into current vaccine candidates include the replacement of two residues with a double proline (e.g., Pfizer/BioNTech [10], Moderna [11], Novavax [13], and Jannsen [14]), as well as mutations in the furin cleavage site for protease resistance (13, 14). The efficacy of these vaccines varies from 62 to 90% for the AstraZeneca/Oxford vaccine to 95% for the mRNA-based platforms of Moderna and Pfizer/BioNTech against the original lineage of SARS-CoV-2 (10, 15, 16). However, recently emerged variants of concern (VOCs) first reported in the United Kingdom (B.1.1.7), South Africa (B.1.351), and Brazil (P.1) are partially resistant to neutralizing antibodies generated against approved vaccines based on the S protein from 2019 strains (17–21). A resulting drop in efficacy has been noted in multiple clinical trials, particularly against lineages containing the E484K mutation (22–24).

An increased stability of S may have advantages in terms of the strength of neutralizing antibody response, particularly in generating antibodies against conformational rather than linear epitopes. *Ex vivo*, a more stable S may facilitate storage and distribution of protein or VLP vaccines to vaccination sites where cold-chain maintenance is difficult. Krammer and coworkers recently compared the immune response in mice expressing combinations of double proline and cleavage site mutants, finding that both were needed in a recombinant protein vaccine to give complete protection against challenge in the mouse model (25).

Sera from infected individuals contain antibodies against S. Neutralizing antibodies, in particular those against RBD, are being evaluated as antibody therapeutics (26). Antibodies against RBD include those that directly block the interaction between the RBD and ACE2, some of which are able to bind to RBD in both up and down conformations, and others which only bind the up conformation (27). Some of these antibodies bind between down RBDs in the same trimer, stabilizing the closed form of the spike. Other antibodies against the RBD bind outside the RBS. Within S there are other epitopes targeted by neutralizing antibodies in the NTD and elsewhere (28, 29). Polyclonal antibody responses that target divergent epitopes are more robust against escape (30).

We and others have developed S protein constructs which exclusively adopt closed prefusion conformations (31–33), where the RBD should not be accessible to ACE2 binding. Preventing transition to an open state leads to an increase in thermal stability. We speculated that closed spikes would lead to a different polyclonal antibody response compared to standard S protein constructs, for example, by driving the maturation of antibodies that lock the RBD in the closed prefusion conformation.

Here, we have immunized mice with a range of different S protein constructs that differ in the presence or absence of the double proline mutant, the mutation present at the furin cleavage site, and in whether they are stabilized in the closed prefusion conformation. Comparison of the resulting immune responses revealed that the closed, stabilized spike induces potently neutralizing antisera that target a different pattern of epitopes compared to more standard stabilized S proteins. These observations suggest that S trimers stabilized in the closed state may have value as a component of the next generation of SARS-CoV-2 vaccines. Such vaccines will be designed to induce a broader spectrum of more potent neutralizing antibody responses of different classes with the aim of generating responses effective in protection from existing and evolving VOCs.

## RESULTS

### Overview of stabilized S protein trimers.

**FIG 1:**
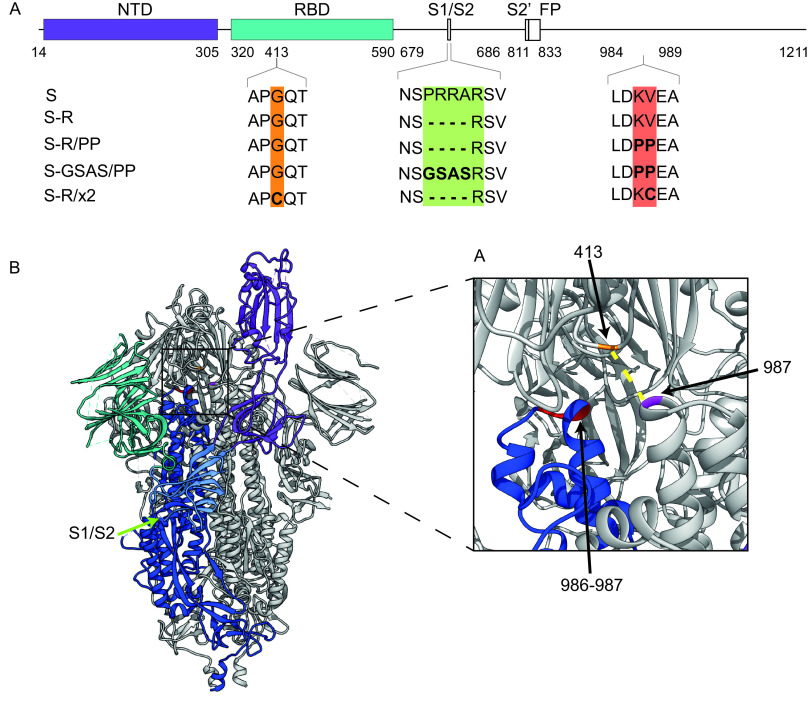
Design of constructs. (A) Overview of constructs used in the study indicating the positions where residues have been mutated. (B) Overview of the trimeric spike structure indicating the positions of the mutated residues. The insertion of cysteine residues at positions 413 and 987 leads to the formation of a disulfide bond (dotted yellow line in inset) that stabilizes S in the closed prefusion form.

We speculated that immunizing with S proteins stabilized in a closed, trimeric conformation would lead to immune responses that differed in strength, and in the range of epitopes targeted, compared to nonstabilized S trimers, or S trimers that are able to transition into the open form. We therefore set out to compare the immune responses to four constructs: S-GSAS/PP, S-R/PP, S-R, and S-R/x2 (Fig. 1A). S-GSAS/PP contains a GSAS sequence preventing cleavage at the furin cleavage site, as well as two stabilizing prolines (4), and provides protection against SARS-CoV-2 infection in mice (25). It is the S antigen of the adenovirus-expressed Janssen vaccine candidate currently in advanced clinical trials (34) and is the protein component of a vaccine under development by Novavax (13). S-R/PP and S-R (31) both have a deletion at the furin cleavage site leaving only a single arginine residue to prevent furin cleavage. S-R/PP additionally contains the two stabilizing prolines. S-R/x2 contains two cysteine residues at positions 413 and 987 which form a disulfide bond that constrains the trimer in its closed state (Fig. 1B) and which results in a dramatic improvement in trimer stability (31). All constructs allow expression and purification of uncleaved S ectodomain trimers (4, 31).

S trimer ectodomains were expressed as described previously (31). Proteins were purified by metal affinity chromatography as described previously (31) and quality controlled by negative stain electron microscopy.

### S-R/x2 is stabilized in a closed state, leading to reduced ACE2 binding and reduced infectivity.

**FIG 2:**
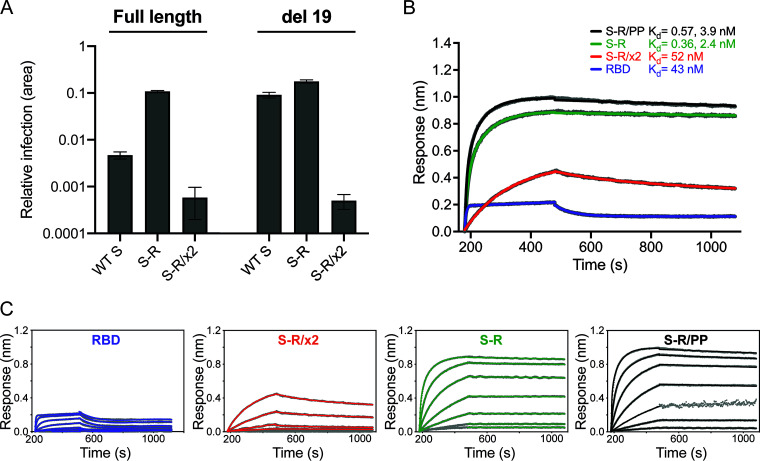
Characterization of fusogenicity and ACE2 binding. (A) Infection of HEK293T-hACE2 cells with SARS-CoV-2 S-pseudotyped HIV virions carrying a GFP reporter gene. The relative area of infected cells was quantified by GFP fluorescence 48 h postinfection using an Incucyte S3 live cell imager. Viruses were produced using either full-length S constructs or a C-terminal deletion of 19 amino acids (del 19) to increase infectivity. Infections were carried out using quantities of virus containing equivalent amounts of S. Virions pseudotyped with wild-type (WT) S are compared to those pseudotyped with S-R and S-R/x2. (B) Biolayer interferometry sensograms for binding kinetics of ACE2 to 600 nM RBD (magenta) and 1,000 nM S-R/PP (black), S-R (green), and S-R/x2 (red). The data are shown in gray with fits to the data in their respective colored lines. The dissociation constants (*K_d_*) shown were calculated from panel C. (C) Concentration series sensograms with fits of the association (*k*_on_) and dissociation (*k*_off_) constants, where *K_d_* = *k*_off_/*k*_on_. The data are summarized in Table 1.

To assess the impact of the disulfide bond in S-R/x2 on S-mediated viral entry, we infected HEK293T-hACE2 cells with S-pseudotyped, replication-deficient lentiviruses, equalized for S incorporation. Pseudovirions bearing S-R/x2 showed minimal infection compared to pseudovirions bearing wild-type S or S-R (Fig. 2A). Deletion of the C-terminal 19 amino acids from S substantially increased infection by pseudovirions bearing wild-type S, consistent with previous data for SARS-CoV-1 (35), and marginally increased the infectivity of S-R pseudovirions. However, pseudoviruses with C-terminally deleted S-R/x2 gave minimal infection (Fig. 2A). These data demonstrate that stabilization of S in the closed state via the x2 disulfide renders the virus largely noninfectious, consistent with the RBS in the RBD being inaccessible for ACE2 binding in the closed state.

We next investigated how stabilizing S in the closed state affected the kinetics of the interaction with ACE2 using biolayer interferometry (Fig. 2B and Table 1). Free RBD associated and dissociated relatively rapidly with a binding constant of 43 nM consistent with previous observations (4). S-R and S-R/PP displayed more complex kinetics, with two association phases: one in the same order as free RBD and one slower, suggesting either different conformations of S or a secondary rate-limiting step such as a conformation change. The dissociation rate was 3 orders of magnitude slower than free RBD, leading to a half-life of the complex of between 2.5 to 5 h and resulting in apparent binding constants in the low-nM and high-pM ranges (Table 1). The low dissociation rate is likely due to avidity from the three RBDs on the S trimer. The interaction kinetics of S-R/x2 with ACE2 were significantly altered with a single association rate 2 orders of magnitude slower than free RBD and the other S proteins and a dissociation rate an order of magnitude faster than the other S proteins. The change in interaction kinetics results in the formation of fewer S-ACE2 complexes (approximately half the amplitude observed for the other S proteins), and the half-life of complexes is reduced to ∼35 min. Hence the binding affinity of S-R/x2 to ACE2 is between 10 and 100 times weaker than the other S proteins, consistent with the ACE2 binding site being largely hidden in S-R/x2.

**TABLE 1:** Summary of fitted association and dissociation constants for the ACE2 interactions^*a*^

Ligand	Analyte	*k*_on_ (M^−1^ s^−1^)	*k*_off_ (s^−1^)	*K_d_*^kin^ (nM)
ACE2-Fc	RBD	3.8 × 10^5^	0.016	43
	S-R/x2	6.3 × 10^3^	3.3 × 10^−4^	52
	S-R	1.0 × 10^5^ (fast)	3.6 × 10^−5^	0.36
		1.5 × 10^4^ (slow)		2.4
	S-R/PP	1.3 × 10^5^ (fast)	7.4 × 10^−5^	0.57
		1.9 × 10^4^ (slow)		3.9

a Fitted association (*k*_on_) and dissociation (*k*_off_) constants are shown. *K_d_*^kin^, the kinetic dissociation constant, is equal to *k*_off_/*k*_on_ from concentration dilution series (see Fig. 2).

### Immunization.

**FIG 3:**
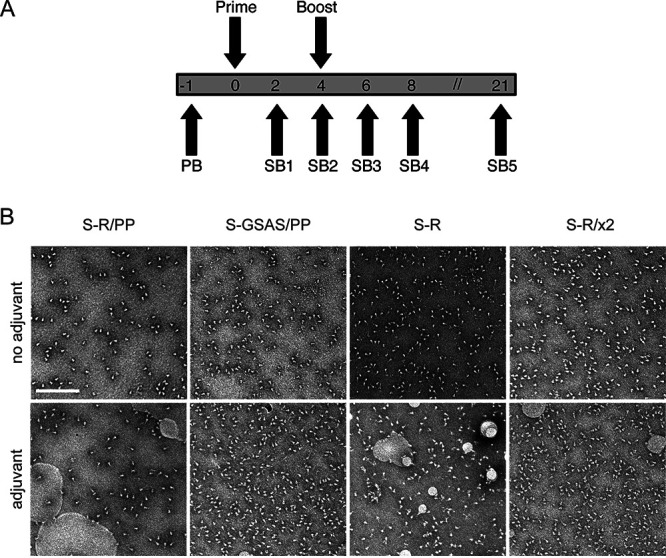
Immunization strategy and proteins for immunization. (A) Overview of immunization strategy. Eight- to ten-week-old BALB/c females were immunized twice with a 4-week interval. Mice were bled 1 week prior to immunization (PB), and serial bleeds (SB) were taken at 2-week intervals after the first immunization, and again at 21 weeks, after which animals were sacrificed and spleens taken for T-cell assays. (B) Oligomeric state of S proteins before and after addition of adjuvant assessed by negative-stain EM. In all cases, the proteins are predominantly in the prefusion, trimeric state. Scale bar, 200 nm.

S trimers were thawed 30 min prior to immunization and were mixed with the adjuvant monophosphoryl lipid A (MPLA) in phosphate-buffered saline (PBS). Mice were immunized with 10 μg of protein mixed with 10 μg of MPLA and then were boosted 4 weeks after the first immunization with 10 μg of protein with 2 μg of MPLA, according to the protocol illustrated in Fig. 3A. Mice were bled 2, 4, 6, 8, and 21 weeks postimmunization (Fig. 3A) and culled 25 weeks postimmunization by cardiac puncture under terminal anesthesia. Adjuvant-mixed trimers were imaged by negative-stain electron microscopy (EM), and in all cases large numbers of trimeric S proteins were visible, validating that S trimers were intact in the presence of adjuvant at the point of immunization (Fig. 3B).

### Trimeric S constructs induce neutralizing sera in mice.

**FIG 4:**
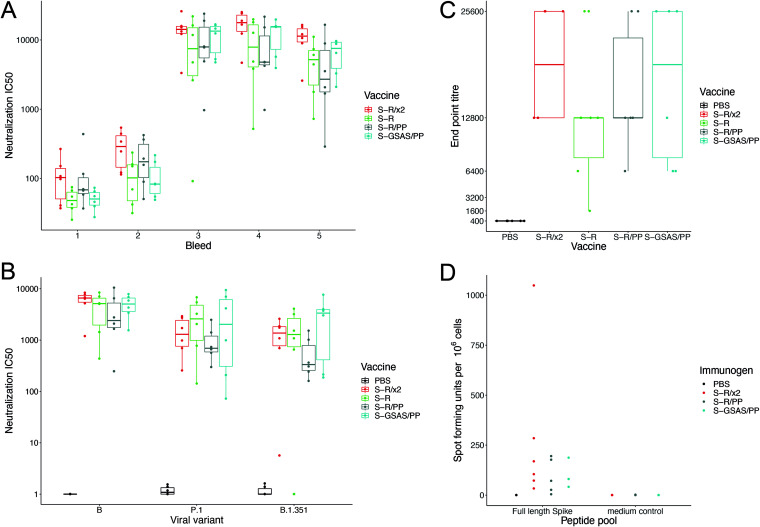
Neutralization by sera and T-cell response. (A) SARS-CoV-2 pseudovirus neutralization IC_50_ values for individual mice at five bleed points postimmunization with one of four S protein variants. The bleed time points are 2, 4, 6, 8, and 21 weeks postprime. (B) SARS-CoV-2 pseudovirus neutralization IC_50_ values for individual mice at 21 weeks postprime using pseudoviruses expressing original S (from 2019 SARS-CoV-2 lineage B) or from VOCs. (C) Box-and-whisker plots for endpoint antibody titers representing the serum dilution point at which the cytopathic effect in Vero cells caused by SARS-CoV-2 BetaCoV/Australia/VIC01/2020 infection was no longer inhibited, compared to controls. Endpoint titer means ranged between 12,800 and 25,600 for vaccine sera. (D) Comparison of persisting T-cell responses 21 weeks postvaccination with stabilized S constructs. T-cell responses were generated by all constructs with some heterogeneity: the SFU range from 0 to 88 for S-R/PP.13, to 79 for S-GSAS/PP, and to 31 to 424 for S-R/x2.

We assessed the degree to which sera were able to neutralize S-mediated infectivity in a lentiviral pseudovirus assay. The majority of mice immunized with S variants had neutralizing sera 2 weeks postimmunization, and by 2 weeks postboost the sera were potently neutralizing, with a geometric mean of 50% pseudovirus neutralization titer for each variant in the range of 7,043 to 12,122 (Fig. 4A). Sera remained potently neutralizing 21 weeks postimmunization. There were no statistically significant differences in the neutralizing titer between the different trimeric S variants assessed at any time point postimmunization; however, the average neutralizing antibody titers for S-R/x2 and S-GSAS/PP were higher than those for S-R and S-R/PP in the functional assays. We also performed neutralization assays using pseudoviruses bearing the spike protein from VOC strains B.1.351 and P.1. These pseudoviruses were still, on average, strongly neutralized by all of the generated antisera; however, several mice showed reductions or ablation of neutralizing antibody titer against either strain (Fig. 4B). These mice showed a variety of different responses in both Luminex binding and surrogate virus neutralization assay inhibition, but no conspicuous pattern can be discerned from the small number of mice in the experiment.

To assess the ability of the sera to neutralize infectious SARS-CoV-2 virus, we performed virus neutralization assays against SARS-CoV-2 BetaCoV/Australia/VIC01/2020 (36) and measured endpoint titers for sera collected at bleed 4 (Fig. 4C). Cytopathic effect-based endpoint neutralizing antibody titers ranged from 1,600 to 25,600 with the S-R/PP average endpoint titer at 16,000, S-GSAS/PP at 17,066, S-R at 12,000, and S-R/x2 at 19,200. These are substantially higher than endpoint titers from a standard high-antibody-titer convalescent human sera control (NIBSC 20/130, see Materials and Methods), which ranged from 1,600 to 6,400, with an average of 3,466.

### Trimeric S constructs induce T-cell responses.

To assess whether immunization led to the development of S-protein-specific T cells, we stimulated splenocytes from vaccinated mice with peptide pools covering the complete S protein and analyzed the response using a gamma interferon (IFN-γ) ELISpot assay (Fig. 4D; see also Fig. S3). We analyzed T cells from sets of 3 (or 6) mice immunized with S-R/GSAS, S-R/PP, and S-R/x2. Though low to moderate, T-cell responses to these different forms of S proteins adjuvanted in MPLA were consistently detected in all immunized mice.

### Trimeric S constructs all induce antibodies that block the ACE2-RBD interaction *in vitro*.

**FIG 5:**
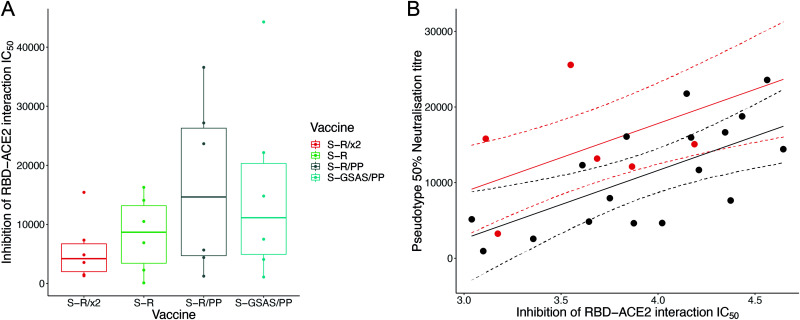
Inhibition by sera of RBD-ACE2 interaction. (A) Boxplot showing IC_50_ values for the surrogate virus neutralization assay that measures the inhibition of RBD-ACE2 interactions by individual mouse sera. (B) Sera from S-R/x2-immunized mice were significantly more potent neutralizers of SARS-CoV-2 pseudovirus than expected based on their direct inhibition of RBD-ACE2 interaction. Predicted neutralizations are shown as a solid line; 95% confidence intervals are shown as dashed lines. S-R/x2 sera and predictions are shown in red, and all other sera are shown in black.

We next explored whether the S protein constructs induced antibodies that are able to directly block the RBD-ACE2 interaction using a surrogate-neutralization assay (GenScript [37]). Sera from bleed 3 were incubated with horseradish peroxidase-coupled RBD (HRP-RBD), which was then bound to ACE2-coated wells before enzymatic readout of the degree of binding. All S-immunized sera inhibited RBD-ACE2 interactions, indicating that both open and closed S protein constructs can induce antibodies that directly block the interaction between ACE2 and the RBS on the RBD (Fig. 5A). These observations are consistent with the exposure of part of the RBS in the closed conformation adopted by the S-R/x2 trimer. We compared the inhibition of RBD-ACE2 interactions with neutralization in the pseudovirus-based assay (Fig. 5B). These variables are well correlated, and this comparison revealed that sera from S-R/x2-immunized mice neutralized virus entry better than would be expected based on their inhibition of the RBD-ACE2 interaction. This observation suggests that the potent neutralization by sera from S-R/x2-immunized mice involves antibodies that prevent infection without directly blocking interaction between the RBS and the receptor. We speculate that such antibodies might include those that bind to the RBD and prevent S opening by conformationally stabilizing the closed state or that bind to other epitopes on S and thereby inhibit receptor interactions or fusion.

### Sera contain antibodies against linear epitopes from surface-exposed positions on S.

**FIG 6:**
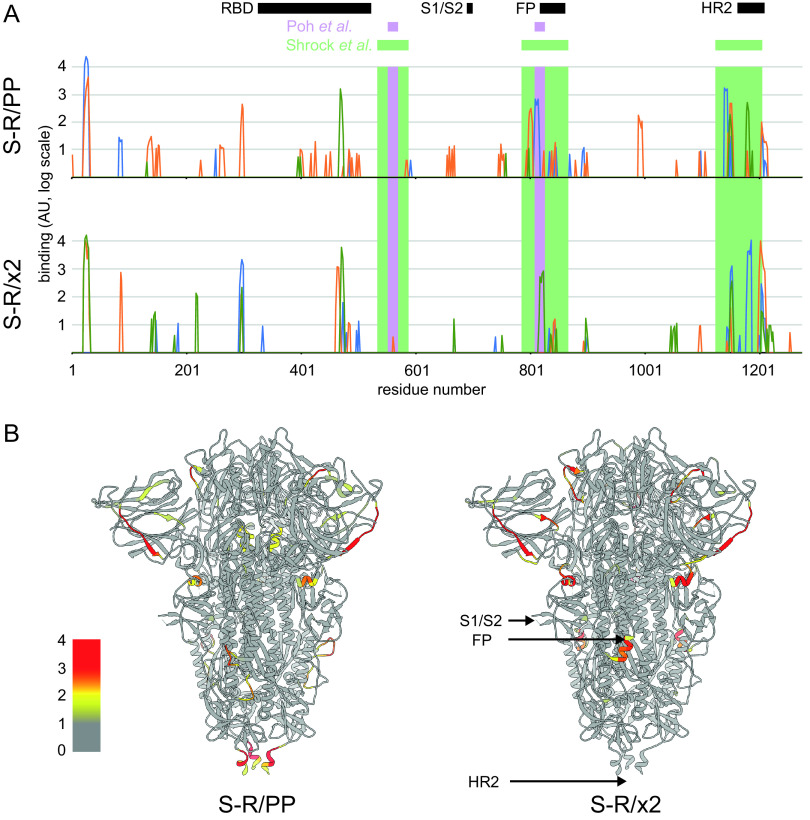
Quantification of binding of sets of three sera immunized with S-R/PP or S-R/x2 to an array of overlapping 15mer linear peptides covering S (see Table S1). (A) The binding strengths of different sera are shown as blue, green, and orange lines. The *x* axis is the residue number at the center of the peptide, and the *y* axis is the intensity of binding shown on a log scale. Different sera bind different patterns of peptides. The positions of the most common linear epitopes identified in human sera by Shrock et al. (38) are shown as pale green rectangles; the positions of two linear peptides that induce a neutralizing antibody response, as identified by Poh et al. (40), are shown as purple rectangles. Structural features are indicated: RBD, S1/S2 cleavage site, fusion peptide (RP), and heptad repeat 2 region (HR2). (B) The positions of the bound epitopes are illustrated on the structure of closed, trimeric S, showing the maximum binding value of the three sera on a log color scale at the center of each peptide (as in panel A) for S-R/PP or S-R/x2. The bound epitopes are almost entirely surface exposed.

For a set of six mice, three immunized with S-R/PP and three immunized with S-R/x2, we performed peptide array analysis using overlapping 15mer peptides covering the complete SARS-CoV-2 S to identify linear epitopes bound by antibodies in the sera (Fig. 6A; see also Table S1). The pattern of linear epitopes varied between sera. They represented almost exclusively surface accessible residues and were predominantly accessible loops or strands (Fig. 6B). Among the most strongly bound epitopes were those in the vicinity of heptad repeat 2 (HR2) and the fusion peptide (FP) which are also widespread in sera from infected humans (38–40). We identified only one strongly binding internal linear epitope, which is centered around residue 991 in a helix, and which bound sera from one of the S-R/PP-immunized mice. This epitope is unlikely to be accessible in the folded prefusion spike, suggesting the presence of some unfolded S-R/PP after immunization.

### Trimeric S constructs all induce antibodies that bind S constructs and RBD, but S-R/x2 immunization leads to different antibodies.

**FIG 7:**
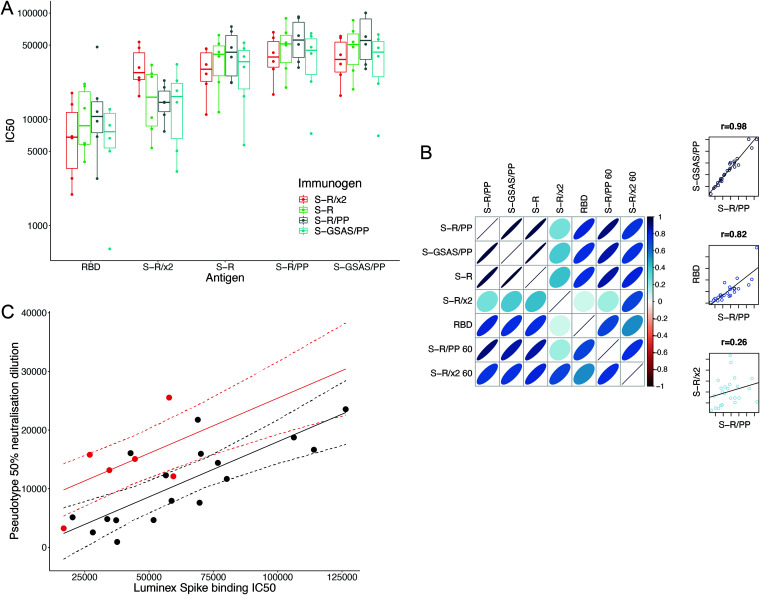
Binding of sera to S protein antigens. (A) IC_50_ values of sera from individual mice immunized with one of four trimeric S constructs binding to five separate antigens. The antigens are SARS-CoV-2 RBD and the four trimeric S constructs. Binding of sera from mice immunized with different S proteins, to different S antigens, was measured using the Luminex assay. All trimeric S constructs induce antibodies that bind all constructs and RBD. Sera from mice immunized with S-R/x2 bound the S-R/x2 antigen more than sera from other mice. (B) Visualization of correlation in IC_50_ values between different antigens. Narrower, darker ellipses indicate stronger correlations. The three scatterplots on the right-hand side are illustrative examples showing the raw relationship for high, low, and intermediate correlations. There is generally a good correlation between the strengths of individual sera binding to different S antigens. This correlation is less strong for RBD and very different for S-R/x2, suggesting that S-R/x2 is displaying different epitopes in this assay than the other S proteins. Heating S-R/x2 leads to it displaying epitopes that are more similar to those of the other S proteins. (C) Sera from S-R/x2-immunized mice were significantly more potent neutralizers of SARS-CoV-2 pseudovirus than expected based on their spike binding IC_50_. Predicted neutralization is shown as a solid line; 95% confidence intervals are shown as dashed lines. S-R/x2 sera and predictions are shown in red, all other sera are shown in black.

Human sera from infected patients contain antibodies against both tertiary and quaternary structural epitopes, as well as the linear epitopes that are represented in peptide arrays. To gain further insights into the sera, we used a multiplexed, particle-based flow cytometry method (Luminex) to measure binding of sera to a range of different S protein antigens: S-GSAS/PP, S-R/PP, S-R, S-R/x2, and RBD (Fig. 7A). Sera bound to all of these antigens. There were no significant differences between binding measured to S-GSAS/PP, S-R/PP, S-R, and RBD for sera from mice that had been immunized with different S proteins. Sera from mice that had been immunized with S-R/x2 did, however, bind more strongly to S-R/x2 antigen than sera from other mice. Within the multiplexed assay, we additionally included S-R and S-R/x2 that had been preincubated at 37°C for 36 h or 60°C for 30 min to induce partial disassembly (see below).

As expected, there is very strong correlation between the binding of individual sera to S-GSAS/PP, S-R/PP, and S-R (*P* ≥ 0.97), reflecting the structural similarities between these antigens (Fig. 7B). There is also substantial correlation between binding to RBD and to these S variants (*P* between 0.75 and 0.8), suggesting that the RBD contains immunodominant epitopes, consistent with observations using human sera (41–43). In contrast, there is poor correlation (*P* < 0.5) between the binding of individual sera to S-R/x2 and to the other S variants or RBD. This suggests that different epitopes are accessible in the S-R/x2 antigen. S-R/x2 that was previously incubated at 60°C for 30 min, which we have previously shown induces partial disassembly (31), binds sera to a degree that correlates better with binding to other S variants (*P* between 0.7 and 0.8) than unheated S-R/x2. This implies that after heating, the S-R/x2 antigen exposes additional epitopes and loses other epitopes in a manner that makes it more similar to the other S antigens. We speculate that heating of S-R/x2 leads to exposure of linear epitopes, exposure of epitopes in the RBD that are hidden in the closed conformation, as well as the loss of structural epitopes that are otherwise best preserved in the more stable S-R/x2 protein.

Sera from S-R/x2 immunized mice show increased binding to S-R/x2 antigen but do not show increased binding to the other S protein antigens. We compared the binding to S-R/PP (as a representative standard S) to pseudovirus neutralization. Sera from mice immunized with S-R/x2 neutralize more strongly than would be predicted based on their binding to S-R/PP (Fig. 7C). Considered together with the observations presented above, these data suggest that immunization with S-R/x2, compared to immunization with other S constructs, leads to a higher fraction of neutralizing antibodies against epitopes which are prevalent in the stable S-R/x2 trimer. We consider it most likely that these are conformational epitopes specific to the closed state. This interpretation explains the observations that S-R/x2 induced sera have increased binding to S-R/x2 antigen and that they neutralize better than expected given their binding to S.

## DISCUSSION

In summary, immunization of mice with trimeric S protein constructs induces very strongly neutralizing sera, and the sera remain neutralizing 5 months postimmunization with neutralizing antibody titers maintained at high levels against the original 2019 SARS-CoV-2 strain and VOCs B.1.351 and P.1. Immunization also induces a T-cell response. All of the sera contain antibodies that bind S and RBD and which block the ACE2-RBD interaction, including sera induced by the stabilized, closed S-R/x2 construct.

The poor correlation between binding of sera to S-R/x2 and to other trimeric S constructs indicates that S-R/x2 displays a different pattern of epitopes when used as an antigen in Luminex assays. Partly this will be due to conformational differences, with S-R/x2 being exclusively in the closed conformation. The observation that heating of S-R/x2 makes it more similar to the other S protein constructs suggests that the S-R/x2 has a more stable quaternary structure than the other antigens, and this more stable structure may also contribute to the different pattern of epitopes.

Our observation that sera induced by the more stable S-R/x2 show significantly better binding to the closed form of the S antigen than sera induced by open or intermediate forms of S trimeric spikes indicates that S-R/x2 raises a different pattern of antibodies compared to other trimeric spikes when used as an immunogen. We suggest that this is for two reasons. First, because of conformational differences: S-R/x2 is exclusively in the closed state, while the other S protein constructs also adopt open states. S-R/x2 therefore does not display epitopes that are hidden in the closed state, particularly some in the RBD. Second, because S-R/x2 is more stable and therefore is likely to remain in a folded state for longer postimmunization; for this reason it may raise more conformational antibodies than S-R. Sera induced by S-R/x2 neutralize better than expected based on their binding to other trimeric spike constructs, suggesting that they indeed contain increased levels of neutralizing antibodies specific to the closed conformation. We therefore conclude that S-R/x2 induces more neutralizing conformational antibodies against the closed state than the other trimeric immunogens.

Here, we have studied the immune response in naive mice that do not express human ACE2. S-R/x2 binds ACE2 with markedly reduced affinity than the other S protein constructs we have assessed. This may lead to enhanced differences when used to immunize species with homologous ACE2 receptors.

On an operational level, the increased thermal stability of S-R/x2 may have important advantages for widespread global distribution, given that cold-chain maintenance is logistically challenging. While we have demonstrated the unique properties of S-R/x2 as a protein immunogen, the S-R/x2 sequence or derivatives thereof can readily be delivered as DNA or RNA as part of next-generation vaccine platforms. The increased thermostability of S-R/x2 may still be useful in genetically encoded vaccines due to persistence of the folded state when the gene is expressed *in vivo*.

New SARS-CoV-2 variants are emerging as the virus evolves increased transmissibility. These include changes in the RBS which increase affinity with human ACE2 and may represent species adaptation. New variants also result from antibody selection pressure. Escape from single neutralizing SARS-CoV-2 monoclonal antibodies to S has been demonstrated *in vitro* (44), and neutralization escape has also been observed in an immunocompromised individual treated with convalescent plasma (45). Virus escape from current S-based vaccines has become a major concern since first-generation vaccine efficacy data are derived from areas where VOCs are spreading in the community. Furthermore, antigen designs to avoid the possibility of inflammatory triggers, such as antibody-dependent enhancement, commonly observed among coronaviruses remain important considerations in vaccinating human populations at risk from severe COVID-19 disease. In the context of immunization of humans previously infected with SARS-CoV-2, or indeed with prior immunization, evaluation of next-generation S antigen candidates to recruit additional beneficial neutralizing responses will be crucial to circumvent a requirement for regular vaccine updates, as needed for other viruses such as influenza. In the light of all these concerns, there is a need to explore more sophisticated immunogens for SARS-CoV-2 vaccination.

The S proteins expressed by the Pfizer/BioNTech and Moderna mRNA vaccines, the AstraZeneca ChAd vaccine, and others are all based on early S protein designs that are able to adopt both open and closed conformations. The S-R/x2 closed spike induces a strongly neutralizing response against a different pattern of epitopes than these constructs. Engineered closed S constructs may therefore be valuable immunogens for inducing neutralizing antibodies against different epitopes. Having S scaffolds such as S-R/x2 that induce polyclonal responses against a different pattern of epitopes allows for flexibility of vaccination regimens at both the individual and the population level.

## MATERIALS AND METHODS

### Protein expression and purification.

All S protein constructs have been previously reported (31). Proteins were expressed and purified exactly as described by Xiong et al. (31).

### Lentiviral pseudotyping to assess infectivity of disulfide-stabilized variant.

Vectors to produce pseudovirions were pCRV-1 (encoding HIV-1 Gag Pol) (46), CSGW (encoding GFP) (47) and pCAGGS-S (encoding spike). pCAGGS-S was generated by cloning of codon optimized S genes into a pCAGGS empty backbone using EcoRI and NheI restriction sites. pCAGGS-S ΔC19 encodes a spike protein lacking the C-terminal 19 amino acids, which have previously been shown for SARS-CoV-1 to contain an endoplasmic reticulum-retention signal (35). Replication-deficient SARS-CoV-2 pseudotyped HIV-1 virions were produced in HEK293T wild-type (293T-wt) cells by transfection with pCAGGS-S, pCRV, and CSGW as described previously (48). Viral supernatants were filtered through a 0.45-μm syringe filter at 48 h posttransfection and pelleted through a 20% sucrose cushion for 2 h at 28,000 rpm. Pelleted virions were resuspended in Opti-MEM.

HEK293T-hACE2 cells were plated into 96-well plates at a density of 7.5 × 10^3^ cells per well and allowed to attach overnight. Lentiviral pseudotype stocks were titrated in triplicate by addition of virus onto cells. Infection was measured by green fluorescent protein (GFP) expression by using an Incucyte live cell imaging system (Sartorius). Infection was enumerated as GFP-positive cell area.

### Biolayer interferometry.

The pCD5-hACE2-Fc expression vector contains the ACE2 ectodomain coding sequence fused at the C-terminal end to a sequence encoding a thrombin cleavage site and a human Fc fragment, as previously described (49). The ACE2-Fc fusion protein was expressed in Expi293 cell and purified from cell culture media using a protein A column, followed by size exclusion chromatography. AceII-Fc at 11 μg/ml was immobilized onto Protein A BioFsensors to a level of ∼0.8 nm in an Octet RED384 (FortéBio). The sensor tips were dipped into 1:3 serial dilutions of RBD or S proteins from initial concentrations of 600 and 1,000 nM, respectively, for 5 min to observe association, followed by transfer to wells containing only assay buffer (10 mM HEPES [pH 7.5], 150 mM NaCl, 3 mM EDTA, 0.05% Tween 20, and 1 mg/ml bovine serum album) to monitor dissociation for 10 min. The assay was run at 30°C. The sensor tips were regenerated with 10 mM glycine (pH 1.5), and the assay was performed again without loading of ACE2-Fc to monitor nonspecific interactions. The data were double referenced, subtracting a buffer reference and the parallel assay without ACE2-Fc. The resultant reference-subtracted data were fitted to single or double phase association and dissociation kinetics to determine *k*_on_, *k*_off_, and *K_d_*^kin^ (the binding constant determined from the ratio of the individual rate constants) using Prism 8.4.3 (GraphPad Software).

### Negative-stain EM.

To characterize protein samples with adjuvant, 10 μg of protein was mixed with 10 μg of monophosphoryl lipid A (MPLA) in a 50-μl final volume of PBS. A portion (3 μl) of sample diluted to 0.05 mg/ml in water was applied to glow discharged (45 s, 30 mA) CF200-Cu carbon film grids and absorbed for 30 s. The grids were side-blotted, washed three times with water, and then stained with Nano-W stain (Nanoprobes), followed by immediate blotting. The grids were air-dried and imaged using a Tecnai T12 microscope operated at 120 kV.

### Animal work.

Eight- to ten-week-old BALB/c females (Charles River) were immunized subcutaneously with 10 μg of purified protein mixed with 10 μg of MPLA in a total volume of 50 μl of PBS. Mice were boosted after a 4-week interval with 10 μg of purified protein mixed with 2 μg of MPLA. Serial bleeds were taken via the saphenous vein at 2-week intervals at D0, D14, D28, D42, D56, and D147. Spleens were removed from mice culled on day 175 for ELISpot analysis.

### Cells and viruses for neutralization assays.

Vero (ATCC CCL 81) and HEK293T/17 (ATCC CRL-11268) cells were cultured in Dulbecco modified Eagle medium supplemented with penicillin (100 U/ml), streptomycin (100 μg/ml), and 10% fetal bovine serum. SARS-CoV-2 (BetaCoV/Australia/VIC01/2020) was obtained from Porton Down, Public Health England, and propagated in Vero cells under BSL-3 conditions. Lentiviral pseudotypes were produced by transient transfection of HEK293T/17 cells with packaging plasmids p8.91 (50, 51) and pCSFLW (52) and a SARS-CoV-2 spike expression plasmid using the Fugene-HD transfection reagent. The ΔC19 spike genes for hCoV-19/Wuhan/IVDC-HB-01/2019 (the initial lineage B strain) or variants P.1 and B.1.351 were codon optimized, synthesized (GeneArt Thermo Fisher, Regensburg, Germany), and cloned into the pEVAC vector using KpnI and NotI restriction sites. Supernatants were taken after 48 h, filtered at 0.45 μm, and titrated on HEK293T/17 cells transiently expressing human ACE-2 and TMPRSS2.

### Pseudotype-based microneutralization assay.

A pseudotype-based microneutralization assay was performed as described previously (53). Briefly, serial dilutions of serum were incubated with SARS-CoV-2 spike-bearing lentiviral pseudotypes for 1 h at 37°C and 5% CO_2_ in 96-well white-cell culture plates. S protein sequences were from hCoV-19/Wuhan/IVDC-HB-01/2019 (lineage B), lineage P.1, or lineage B.1.351. A total of 1.5 × 10^4^ HEK293T/17 cells transiently expressing human ACE-2 and TMPRSS2 was then added per well, and the plates incubated for 48 h at 37°C and 5% CO_2_ in a humidified incubator. Bright-Glo (Promega) was then added to each well, and the luminescence read after a 5-min incubation period. Experimental data points were normalized to 100 and 0% neutralization controls, and nonlinear regression analysis was performed to produce neutralization curves and 50% inhibitory concentration (IC_50_) values.

### Virus neutralization assay.

Vero cells were plated in 96-well clear cell culture plates to reach confluence on the day of infection. Serial dilutions of serum in PBS were incubated with 500 PFU/well of BetaCoV/Australia/VIC01/2020 in PBS for 1 h at 37°C and 5% CO_2_ in a humidified incubator before being transferred to 96-well Vero cell monolayers. Plates were then incubated at room temperature on a rocking platform. After 90 min, 100 μl of 2× DMEM (2% final FBS) was added, and the plates were incubated at 37°C and 5% CO_2_ in a humidified incubator. After 48 h, the medium was removed, and the cells were fixed with 10% formalin for 30 min at room temperature. The cells were then stained with 0.25% crystal violet solution, and the endpoint dilutions were measured.

### Murine IFN-γ ELISpot assay.

Using the flat edge of a 5-ml sterile syringe stopper head, the spleens of freshly sacrificed mice were passed through a 70-μm cell strainer fitted onto a 50-ml Falcon tube. Smashed spleen tissue was then washed down through the cell strainer using PBS warmed to room temperature, and the resulting cell suspension was centrifuged at 330 × *g* for 5 min. To remove red blood cells, the cell pellet was resuspended in 2 ml of PBS, overlaid on an equal volume of Histopaque 1083 (Sigma-Aldrich, 10831-100M), and centrifuged at 400 × *g* for 30 min without brake. The layer of white cells was carefully extracted and washed at 330 × *g* for 5 min in RPMI 1640 (Gibco, catalog no. 21875-034) prewarmed to 37°C. The cell pellet was resuspended in prewarmed (37°C) complete CTL-Test medium, and live cells were counted. The cell concentration was adjusted to 4 × 10^6^ cell/ml and stored in a humidified incubator at 37°C and 5% CO_2_ prior to performing the ELISpot assay.

The ELISpot assay was performed using a murine IFN-γ single-color enzymatic ELISPOT assay kit (CTL). Briefly, polyvinylidene difluoride membranes were coated with IFN-γ capture antibody overnight and then washed with PBS. Next, 100 μl of test peptide (at a final concentration of 1 μg/ml) PepMix SARS-CoV-2 S (JPT, PM-WCPV-S-1) or murine anti-CD3 antibody (positive control; Invitrogen, catalog no. 16-0031-82) or CTL-Test medium (negative control) was applied to the wells, and the plates were then placed in a humidified incubator at 37°C and 5% CO_2_. Splenocytes were plated at 4 × 10^5^ cells/well in 100 μl of suspension in CTL-Test medium and immediately transferred to a humidified incubator at 37°C and 5% CO_2_ for 24 h. After development of spot-forming units (SFU) and prior to analysis, the plates were dried overnight. The plates were scanned and analyzed using an ImmunoSpot S6 Ultra-V analyzer (CTL). For all wells, the numbers of SFU were determined using the SmartCount and Autogate and are expressed as SFU/million cells. Both peptide stimulation and medium treatment were carried out in triplicate for each sample.

### SARS-CoV-2 surrogate virus neutralization test.

Blocking of the RBD-ACE2 interaction by the mouse sera was assessed using a SARS-CoV-2 surrogate virus neutralization test kit (GenScript) (37) according to the manufacturer’s instructions. Briefly, sera from bleed 3 were diluted in PBS, before further dilution in the provided sample buffer at a 1:9 ratio. Samples and controls were then mixed with HRP-RBD, incubated in 37°C for 30 min, and added to the ACE2-coated wells. The plate was incubated at 37°C for 15 min and washed four times. TMB solution was added to the reactions, followed by a 15-min incubation in the dark at room temperature. The absorbance at 450 nm was read immediately following quenching with the provided stop solution (Synergy H1 hybrid multi-mode reader; BioTek). The IC_50_ was estimated using a three-parameter log-logistic regression fit in GraphPad Prism. The absorbance at 450 nm was modeled in response to serum dilution.

### Peptide epitope scanning.

Peptide epitope scanning was performed against a microarray of 15mer peptides with 13mer overlaps covering the SARS-CoV-2 spike protein, as well as other coronavirus spike proteins using the PEPperCHIP Pan-Corona spike protein microarray (54, 55). Experiments were performed by PEPperPRINT GmbH (Heidelberg, Germany). Prestaining of the peptide microarray was done with the secondary antibody to identify any background interactions with the 4,564 different spike protein peptides of the microarray that could interfere with the main assays. Subsequent incubation of other microarrays with mouse sera at 1:100 dilution was followed by staining with secondary and control antibodies. Readout was performed with a LI-COR Odyssey imaging system at scanning intensities of 7/7 (red/green). Additional HA control peptides framing the peptide microarrays were subsequently stained as internal quality control to confirm the assay performance and the peptide microarray integrity. Quantification of spot intensities and peptide annotation were performed with a PepSlide analyzer (PEPperPRINT GmbH). In Fig. 6, spot intensities were plotted on a log_10_ scale.

### Multiplex particle-based flow cytometry (Luminex).

Luminex assays were performed essentially as previously described in Xiong et al. (31). RBD, S-GSAS/PP, S-R/PP (two independent preparations), S-R, and S-R/x2, as well as S-R and S-R/x2 preincubated at 37°C for 30 min or 60°C for 30 min, were covalently coupled to distinctive carboxylated bead sets (Luminex; Netherlands) to form a 10-plex assay.

The S-variant and RBD coupled bead sets were incubated with sera from immunized mice at four dilutions (1:100, 1:1,000, 1:10,000, and 1:100,000) for 1 h in 96-well filter plates (MultiScreenHTS; Millipore) and analyzed on a Luminex analyzer (Luminex/R&D Systems) using Exponent Software v31. Specific binding was reported as mean fluorescence intensities (MFIs). The MFI values at each of the four dilutions were used to estimate IC_50_ values as described below.

### Statistical tests.

Pseudovirus neutralization and antigen binding potency were estimated using a four-parameter log-logistic dose-response curve. Dose response curves were fit using the “drc” (56) package in R (57). A single model was fit to data from all mice for a given experiment, but a separate IC_50_ value was estimated for each mouse. This meant that a separate curve was fit to each mouse, but they only differed in their IC_50_s.

To test whether S-R/x2 sera neutralized pseudovirus better than expected given its S-R/PP binding or surrogate virus neutralization, we fit linear regression models predicting pseudovirus neutralization based on S-R/x2 status and either S-R/PP binding IC_50_ or surrogate virus neutralization IC_50_. In these models, S-R/x2 status was a binary variable indicating if the mouse was immunized with S-R/x2 or not. The significance of the S-R/x2 status was assessed by analysis of variance with a simplified model where the S-R/x2 status was not included. These models and comparisons were performed in R (57).
